# Predictive value of free fatty acid levels in embolic stroke of undetermined source: A retrospective observational study

**DOI:** 10.1097/MD.0000000000022465

**Published:** 2020-10-02

**Authors:** Mi-Yeon Eun, Joo Hye Sung, Sang-Hun Lee, Ileok Jung, Moon-Ho Park, Yong-Hyun Kim, Jin-Man Jung

**Affiliations:** a Department of Neurology, Kyungpook National University Chilgok Hospital, School of Medicine, Kyungpook National University, Daegu; b Department of Neurology, Korea University Anam Hospital, Korea University College of Medicine, Seoul; c Department of Neurology, Korea University Ansan Hospital, Korea University College of Medicine; d Department of Neurology, Ho-one Geriatric Hospital; e Department of Cardiology, Korea University Ansan Hospital, Korea University College of Medicine; f Korea University Zebrafish Translational Medical Research Center, Ansan, Republic of Korea.

**Keywords:** diastolic blood pressure, embolic stroke of undetermined source, free fatty acid, left atrial volume index, potential embolic sources

## Abstract

The present study aimed to investigate the predictive value of free fatty acid (FFA) in embolic stroke of undetermined source (ESUS) according to the presence of potential embolic sources (PES) after extensive etiologic evaluation.

This was a retrospective observational study based on a single-center registry from January 2011 to July 2017. Stroke subtypes were determined through laboratory findings, brain, and angiographic imaging, carotid ultrasonography, transthoracic echocardiography, and 24-hour Holter monitoring. If ESUS was suspected, transesophageal echocardiography was additionally performed. Patients were classified into ESUS with PES and ESUS without PES. PES included mitral annular calcification, mitral valve prolapse, patent foramen ovale, atrial septal aneurysm, spontaneous echo contrast, ventricular aneurysm, and high-risk plaques of aortic arch, or carotid bulb. We compared clinical and laboratory findings between the two groups.

Of a total of 110 ESUS patients, 61 patients (55.5%) had no PES. Patients with ESUS without PES had higher levels of serum FFA, systolic blood pressure, diastolic blood pressure (DBP), and left atrial (LA) enlargement compared with those of ESUS with PES. Multivariable analysis demonstrated that the FFA level, DBP, and LA volume index were associated with ESUS without PES [odds ratio (OR) 1.038, 95% confidence interval (CI) 1.019–1.058 for FFA/10 μEq/L, OR 1.414, 95% CI 1.037–1.928 for DBP/10 mm Hg, and OR 1.073, 95% CI 1.009–1.141 for LA volume index].

Higher levels of FFA, DBP, and LA volume index are associated with ESUS without PES, highlighting the need to identify the role of these markers in ESUS through further large-scale, multi-center and prospective studies.

## 1 Introduction

Approximately one-fourth of all ischemic strokes remain undetermined etiology, and stroke without identified cause even after the thorough diagnostic evaluation is classified into the cryptogenic stroke.[^1^,^2^] However, the definition of cryptogenic stroke varies according to the studies. As most cryptogenic strokes are presumed to have a thromboembolic mechanism, the terms of “embolic stroke of undetermined source (ESUS)” were introduced.^[3]^ ESUS may be originated from various potential embolic sources (PES) including minor-risk cardiac sources, arterial plaques or paradoxical emboli. In addition, covert paroxysmal atrial fibrillation (AF) and atrial cardiopathy may be conceived as one of the important causes of ESUS without other embolic sources.^[3]^

Identifying etiology in patients with ESUS is important for therapeutic implications. Recent randomized controlled trials failed to prove the superiority of direct oral anticoagulants compared with aspirin for preventing recurrent stroke in patients with ESUS.[^4^,^5^] The crucial reason is the heterogeneity of embolic sources (arterial, cardiogenic, or paradoxical) leading to ESUS. The most compelling case of anticoagulation may be that of ESUS related to covert AF. In recent studies, prolonged cardiac rhythm monitoring could identify more paroxysmal AF than standard monitoring in patients with stroke.[^6^,^7^] However, it is challenging for applying all patients with ESUS in real-world practice due to great expense and inadequate patient's compliance. Moreover, the detection rate for covert AF did not exceed 30% even after extended monitoring.^[8]^ Therefore, it may be beneficial to have a biomarker for covert AF in patients with ESUS. A recent study suggested some coagulation markers and left atrial volume index (LAVI) could predict AF in cryptogenic stroke.^[9]^ In addition, free fatty acid (FFA) is an emerging candidate in AF-related stroke.[^10^,^11^,^12^] FFA is important energy substrates for heart, and increased FFA levels contribute to myocardial dysfunction and arrhythmia.^[13]^ However, the clinical implication of FFA have not been evaluated in patients presented with ESUS.

In the present study, we investigated the association between FFA levels and ESUS according to the presence of PES. We also compared the FFA level of patients with ESUS with those of other stroke subtypes including AF-related stroke.

## 2 Materials and methods

### 2.1 Study design and population

This was a retrospective observational study to assess the clinical implication of FFA on ESUS based on a hospital-based stroke registry. Patients with acute ischemic stroke who were consecutively admitted to Korea University Ansan Hospital from January 2011 to July 2017 were enrolled. The inclusion criteria were as follows:

1) a diagnosis of acute ischemic stroke within 7 days of symptom onset,
2) relevant ischemic lesions on diffusion weighted magnetic resonance (MR) imaging, and
3) presumed ESUS. ESUS was defined as non-lacunar stroke without relevant intracranial or extracranial arterial stenosis (≥ 50%), major cardioembolic sources, and other etiology according to the proposed criteria by the Cryptogenic Stroke/ESUS International Working Group.^[3]^ Those who did not undergo transesophageal echocardiography (TEE) nor had FFA data, were excluded. Included patients were further grouped into ESUS with PES and ESUS without PES.

This study was approved by the Institutional Review Board (AS0685). The need for written informed consent was waived due to the retrospective design and the minimal risk to patients.

### 2.2 Data collection and etiologic evaluation

Information on demographic data, characteristics of stroke (severity and etiology), medical history of risk factors (hypertension, diabetes mellitus, coronary artery disease, and current smoking) was obtained. We performed brain imaging including diffusion-weighted MR imaging, intracranial and extracranial arterial imaging using MR angiography and duplex ultrasound imaging, laboratory tests, and cardiac workup as routine evaluation in all of the included patients. Routine laboratory examinations included complete blood count, electrolyte, glucose, renal function test, liver function test, lipid profile (total cholesterol, low-density lipoprotein cholesterol, high-density lipoprotein cholesterol, and triglyceride), and FFA. Blood samples were acquired after at least 8 hour-fasts the morning after admission. Level of serum FFA was measured by the enzymatic colorimetric method, using the NEFA-HR (2) reagent kit (Wako Pure Chemical Industries, Ltd., Osaka, Japan).^[14]^ In patients < 45 years old with unknown etiology, we performed laboratory assessments for pro-thrombotic state including coagulation factors and anti-phospholipid antibodies.

In principle, stroke subtypes were categorized according to the Trial of Org 10,172 in Acute Stroke Treatment (TOAST) classification.^[15]^ However, cardioembolic stroke was determined as a case accompanied by high-risk cardioembolic sources. For determination and risk stratification of cardioembolic sources, we adopted and modified a list from TOAST and Stop Stroke Study TOAST.^[16]^ Consequently, we defined high-risk cardioembolic sources as follows: bioprosthetic and mechanical valve, rheumatoid mitral, or aortic valve disease, AF (other than loan AF), sustained atrial flutter, sick sinus syndrome, left atrial (LA)/atrial appendage thrombus, left ventricle thrombus, recent myocardial infarction (< 4 weeks), chronic myocardial infarction together with low ejection fraction less than 28%, symptomatic congestive heart failure with ejection fraction less than 30%, dilated cardiomyopathy, akinetic left ventricular (LV) segment, nonbacterial thrombotic endocarditis, infective endocarditis, atrial myxoma, and papillary fibroelastoma.[^15^,^16^] Stroke of undetermined etiology with negative evaluation results or cardioembolic stroke with low or uncertain risk embolic sources were classified as ESUS based on the thorough etiological evaluation.

### 2.3 Cardiac workup and parameters

We conducted electrocardiography, 24-hour Holter monitoring, and transthoracic echocardiography in every patient with ischemic stroke. Besides, at least 48-hour electrocardiography monitoring in stroke unit was applied to identify paroxysmal AF or other arrhythmias since September 2014. If patients were categorized into undetermined etiology, TEE was performed and interpreted by certified echocardiography cardiologists (YHK and SWK) for detection of complex aortic arch atheroma and hidden cardioembolic sources. The type of AF was defined as paroxysmal versus sustained (persistent or permanent).^[17]^ The echocardiographic data were reviewed by using a viewer program (Centricity Enterprise Web, GE Medical Systems). Echocardiographic parameters of interest were LA anterior-posterior diameter, LAVI, ejection fraction, LV diastolic function, inward and outward flow velocities in the LA appendage.

### 2.4 Lesion pattern

Ischemic lesions were divided into embolic versus non-embolic patterns based on initial diffusion weighted imaging. Imaging criteria for embolic stroke patterns referred to at least one of the following; multiple acute infarcts, simultaneous involvement of different circulations (both right and left anterior circulations or both anterior and posterior circulations), multiple infarcts of different ages, or isolated cortical ischemic lesions.[^14^,^18^]

### 2.5 PES

PES include mitral annular calcification, mitral valve prolapse, patent foramen ovale (PFO), atrial septal aneurysm, spontaneous echo contrast, ventricular aneurysm, complex aortic arch atheroma, and high-risk carotid plaques. Spontaneous echo contrast was diagnosed in cases of an echogenic swirling pattern in the left atrium or LA appendage.^[19]^ Complex aortic arch atheromas were diagnosed when their thickness was over 4 mm or complex plaque (mobile or ulcerated) was found.^[20]^ High-risk carotid plaques were defined as non-stenotic carotid plaques with surface ulceration.^[21]^

### 2.6 Statistical analysis

Continuous variables were reported as mean ± standard deviation or median and interquartile range. Simple comparisons of the 2 groups were performed using the *χ* ^*2*^ test for categorical variables and Student's *t*-test or Mann–Whitney *U* test for continuous variables after assessing normality of each of them. The association between the FFA level and ESUS without PES was investigated using univariate and multivariable logistic regression analyses. Multivariable logistic regression models were developed by inputting variables with *P* value < .1 in univariate analyses and performed using backward elimination. All of the analyses were conducted using SPSS 20.0 for Windows (IBM Corporation, Armonk, NY). A 2-sided *P* value < .05 was considered statistically significant.

## 3 Results

**Figure 1:**
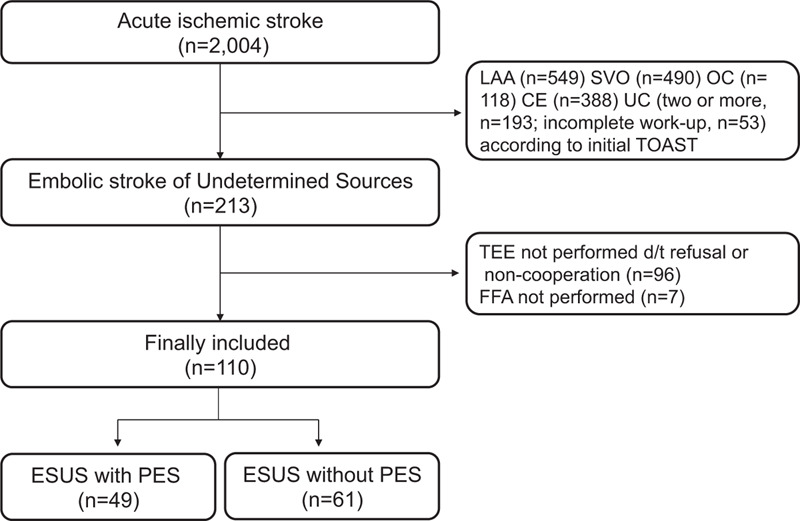
Flowchart of included patients. CE = cardioembolism, ESUS = embolic stroke of undetermined source, LAA = large artery atherosclerosis, OC = other cause, PES = potential embolic sources, SVO = small vessel occlusion, UC = undetermined cause.

Of 2,004 patients, 213 were classified as ESUS. Excluding 96 patients who did not undergo TEE due to noncooperation or refusal and 7 patients without FFA levels or with non-fasting FFA levels, a total of 110 patients were finally included (Fig. 1). There was no missing value in the baseline characteristics in included patients. Among them, 49 patients (44.5%) have PES. Identified PES were PFO (34, 30.9%), complex aortic arch atheroma (16, 14.5%), atrial septal aneurysm (2, 1.8%), mitral annular calcification (2, 1.8%) and high-risk carotid plaques (2, 1.8%). Otherwise, spontaneous echo contrast, mitral valve prolapse, and ventricular aneurysm were absent. The 4 patients (3.6%) have 2 or more PES. Patients without PES were characterized by higher systolic blood pressure, diastolic blood pressure (DBP), and fasting blood glucose compared to those with PES. The FFA level was significantly higher in patients without PES (762 μEq/L, 618.5–1008.5) than patients with PES (532 μEq/L, 351–735.5) (Table 1). LA volume and LAVI tended to be higher in patients with ESUS without PES, but it was not statistically significant. Vascular risk factors, use of antiplatelet agents and statins, and stroke severity were not different between groups.

**Table 1:**
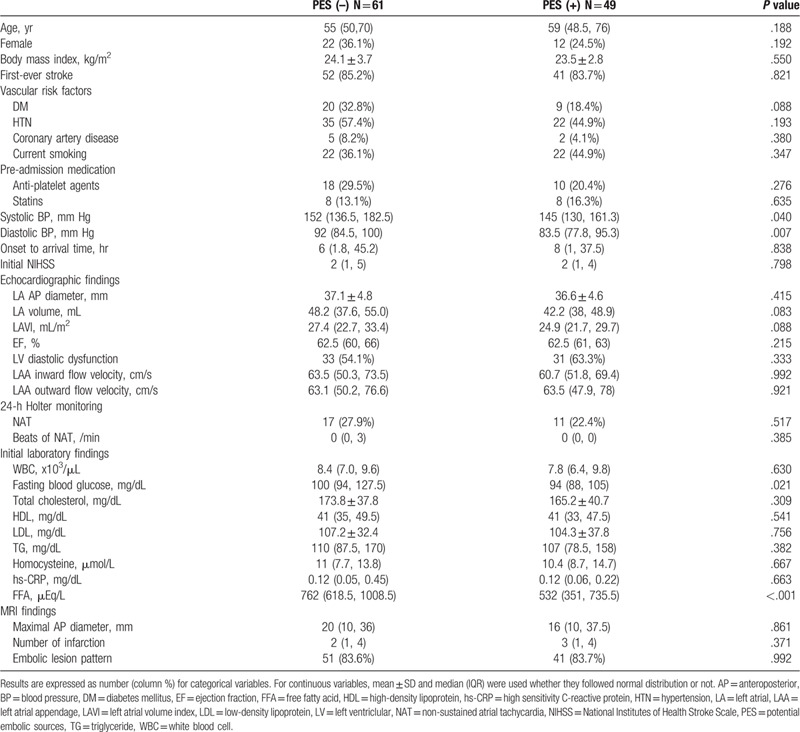
Baseline characteristics of included patients.

A total of 92 patients (83.6%) had embolic lesion pattern (51 patients without PES and 41 with PES). Among them, 26 had multiple lesions in multiple territories, 37 patients had multiple lesions in single territory, and 29 single cortical lesion. With regards to non-embolic lesion pattern, single, and non-lacunar infarction was found in 18 patients.

### 3.1 FFA level predicting for ESUS without PES

Variables for multivariable analysis were selected after univariable analyses (all *P*-values < .1). Multivariable analysis demonstrated that the FFA level, DBP, and LAVI were associated with ESUS without PES (Table 2). The FFA level (/10 μEq/L) was associated with ESUS without PES [odds ratio (OR), 1.038; 95% confidence interval (CI), 1.019–1.058]. The relationship between the FFA level and ESUS without PES showed a level-response relationship. The highest tertile of FFA levels (OR, 11.131; 95% CI, 3.581–34.602) and middle tertile of FFA (OR 4.814; 95% CI, 1.665–13.915) were independently associated with ESUS without PES compared with the lowest tertile.

**Table 2:**
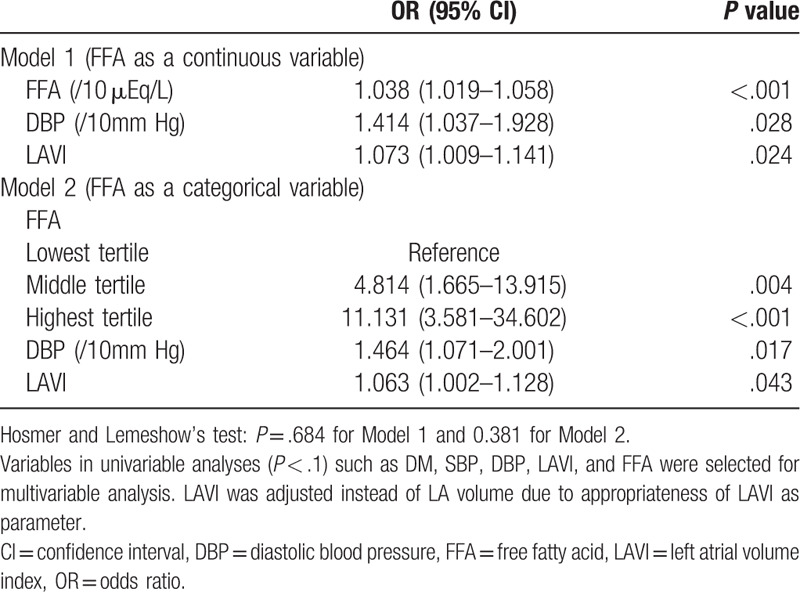
Effects of free fatty acid on predicting embolic stroke of undetermined source without potential embolic sources.

In addition, multivariable analyses demonstrated that the FFA level (OR 1.032, 95% CI 1.013–1.052 for FFA/10 μEq/L) and LAVI (OR 1.07, 95% CI 1.003–1.142 for LAVI) are related to ESUS without PES in 92 patients with embolic lesion patterns.

### 3.2 Comparison of the FFA level of ESUS with those of other stroke subtypes

Of 2004 whole population, FFA levels were extracted according to stroke subtypes (large artery occlusion and small vessel occlusion) and type of AF in cardioembolic stroke. Table 3 demonstrated the comparison between these groups. The level of FFA in patients with sustained AF was the highest, and that of paroxysmal AF was next. Interestingly, the FFA level in the ESUS without PES was similar to that of the paroxysmal AF-related stroke group and higher than stroke of large artery atherosclerosis and small vessel occlusion. The FFA level of the ESUS with PES was lower than that of ESUS without PES.

**Table 3:**
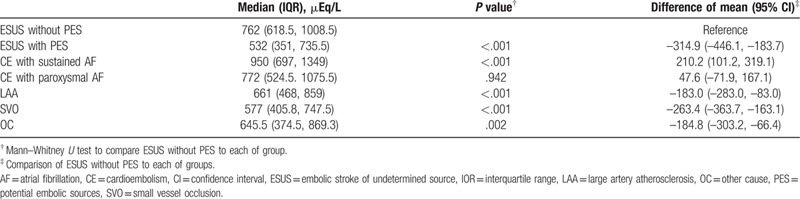
Comparison of free fatty acid levels according to stroke subtypes.

## 4 Discussion

In our study, PES were not found in approximately half (55.5%) of patients with ESUS, even after extensive etiologic evaluation including TEE. High levels of FFA, LAVI, and diastolic BP were associated with ESUS without PES after adjusting other confounding factors including clinical, laboratory, and echocardiographic findings. The relationship strengthened proportionally with increases of the FFA level. Furthermore, FFA was more elevated in ESUS without PES than ESUS with PES and stroke of large artery atherosclerosis and small artery occlusion. Interestingly, the level of FFA in ESUS without PES were similar to those of stroke with paroxysmal AF.

Biomarkers can be useful for predicting etiology and prognosis in acute ischemic stroke.[^9^,^22^] Our study demonstrated that the level of FFA predicted ESUS without PES. Recently, there is growing evidence that FFA is an emerging biomarker of stroke with embolic sources of cardiac origin. Elevated FFA induces inflammation^[23]^ and several inflammatory markers were related to cardioembolic stroke mainly composed of AF.[^24^,^25^] Furthermore, recent observational studies have shown that the FFA level was associated with ischemic stroke attributed to AF.[^11^,^12^,^14^] Although we did not corroborate the direct evidence of covert AF in these patients through prolonged monitoring, the increased FFA levels in those may imply the presence of covert AF.

In particular, the similar level of FFA in ESUS without PES and ischemic stroke with paroxysmal AF supported reasonable suspicion about this inference. Another possibility was that the level of FFA might be related to novel cardioembolic sources such as atrial cardiopathy. Finally, FFA may constitute one of the risk factors for ESUS without PES via platelet aggregation and hypercoagulability.[^26^,^27^]

If the elevated level of FFA were suggestive of covert AF in patients with ESUS without PES, the level of FFA in patients with ESUS without PES could reflect the future risk of recurrent stroke. Previous observational study demonstrated that FFA levels could predict recurrent stroke in patients with AF-related stroke after adjustment for established risk scoring system.^[10]^ One of the plausible mechanisms of the predictive value of FFA on the outcome of AF-related stroke is ascribed to the thrombotic effect of FFA. The other explanation was based on the results of our study. Patients with sustained AF had higher levels of FFA than those with paroxysmal AF. In other words, FFA levels seemed to reflect the AF burden. Several clinical trials have shown that AF burden was related to the risk of stroke.[^28^,^29^]

Interestingly, the FFA level of ESUS without PES were higher than those of ESUS with PES. Some embolic sources were related to FFA. Seo et al reported the relationship between the FFA level and cardioembolic sources such as AF, valvular heart disease, congestive heart failure with low ejection fraction, LV thrombus, LA thrombus, and LV wall motion abnormality.^[14]^ However, FFA did not predict the low-risk cardioembolic sources of PFO and mitral annular calcification. Besides, only AF showed the definite level-response relationship to FFA. Thus, the heterogeneous impact of FFA on embolic sources and the strongest association with AF can account for this finding.

In addition to FFA, LAVI was independently associated with ESUS without PES. LA enlargement interplays with AF, as a potent risk factor and the consequence.[^30^,^31^] On the other hand, moderate to severe LA enlargement was an independent risk factor for recurrent cardioembolic or cryptogenic stroke, suggesting that LA enlargement may be the common pathomechanism in these stroke subtype.^[32]^ Atrial dysfunction represented by LA enlargement could lead to stasis, endothelial dysfunction, and thromboembolism even in the absence of AF. Considering the reciprocal association between AF and LA enlargement, a major culprit in ESUS without PES may be a covert paroxysmal AF and atrial cardiopathy.

We found that DBP also predicted independently ESUS without PES. Elevated blood pressure is one of the most powerful risk factors for stroke. Although several observational studies demonstrate that DBP was related to peri-procedural stroke, systolic blood pressure usually has a greater effect on the risk of stroke.[^33^,^34^] The role of DBP in ESUS without PES needs to be elucidated.

Our study has strengths of extensive evaluation including TEE, 24-hour Holter monitoring, and laboratory evaluation for thrombophilia especially in stroke at young age. The extent of mandatory workup in patients with ESUS is not fixed. However, several organizations recommend TEE for cardiac sources of embolism in patients with no identified embolic sources or highly suspected.[^35^,^36^] TEE is the gold standard of identifying some of the embolic sources (e.g., PFO, aortic arch atheroma) and may decisively affect the etiologic classification and therapeutic strategies.^[37]^ Therefore, our results could precisely demonstrate the association with FFA and ESUS according to the presence of PES.

We have to mention some limitations of our study. First, the results of the current study may not be generalizable because they are findings from a small number of retrospective registry of a single center in Korea. However, we can perform more extensive evaluations in all patients under the same conditions. Second, there might be a selection bias because we included only patients who underwent TEE. TEE was performed in a small number of patients who agreed and were able to cooperate. Third, we did not acquire further information about atrial cardiopathy, such as atrial morphology through cardiac MR, various inflammatory cytokines, or LA dysfunction. Finally, we did not conduct prolonged and/or invasive cardiac monitoring to confirm that the higher level of FFA in ESUS without PES were correlated with detection of covert AF. However, prolonged monitoring is not practical in the field of real-world clinical practice, and also there is no consensus on the duration of monitoring. Therefore, our results should be interpreted cautiously.

In conclusion, the level of FFA and DBP, and LAVI are associated with ESUS without PES, highlighting that further large-scale, multi-center and prospective studies are warranted to affirm the role of these markers in ESUS.

## Acknowledgments

We thank Jaehyung Cha, a statistician from Medical Science Research Center, Korea University Ansan Hospital, for all his efforts in performing and recommending for data analysis. We also thank for performing and interpretation of echocardiography, by Prof. Sunwon Kim, Department of Cardiology, Korea University Ansan Hospital.

## Author contributions

**Conceptualization:** Mi-Yeon Eun, Joo Hye Sung, Jin-Man Jung.

**Data curation:** Joo Hye Sung.

**Formal analysis:** Joo Hye Sung, Jin-Man Jung.

**Funding acquisition:** Jin-Man Jung.

**Investigation:** Sang-Hun Lee, Ileok Jung, Moon-Ho Park, Jin-Man Jung.

**Methodology:** Jin-Man Jung.

**Supervision:** Yong-Hyun Kim, Jin-Man Jung.

**Visualization:** Mi-Yeon Eun, Joo Hye Sung, Jin-Man Jung.

**Writing – original draft:** Mi-Yeon Eun, Joo Hye Sung.

**Writing – review & editing:** Mi-Yeon Eun, Jin-Man Jung.
